# Plasma concentration of interleukin-6 was upregulated in cancer cachexia patients and was positively correlated with plasma free fatty acid in female patients

**DOI:** 10.1186/s12986-019-0409-9

**Published:** 2019-11-15

**Authors:** Jun Han, Chaocheng Lu, Qingyang Meng, Alice Halim, Thong Jia Yean, Guohao Wu

**Affiliations:** 1 Department of General Surgery, Zhongshan Hospital of Fudan University, 180 Fenglin Road, Shanghai, 200032 China; 2 Shanghai Medical College, Fudan University, 138 Yixueyuan Road, Shanghai, 200032 China

## Abstract

**Background:**

Cancer cachexia is a clinical manifestation in various advanced cancers that characterized by muscle atrophy and fat loss as its main features; it is frequently associated with systemic inflammatory response. However, the differences in inflammatory response and lipid metabolism of different genders remain unclear. This study explores the difference between cachexic and non-cachexic patients in different genders and cancer types and focus on the plasma inflammation factors levels and lipid metabolism parameters in different genders.

**Methods:**

We first analyzed the general characteristics in 311 cancer patients between cachexic and non-cachexic patients, with an emphasis on expression levels related to inflammatory factors and lipid metabolism parameters. We then further analyzed these characteristics in different genders and cancer types. Lastly, the correlations between plasma interleukin-6 (IL-6) and lipid metabolism parameters in cachexia patients of different genders were analyzed.

**Results:**

Among 311 patients, there were 74 cancer cachexia patients (50 males and 24 females) and 237non-cachexia patients (150 males and 87 females). Body mass index (BMI), TNM stage, plasma concentration of hemoglobin, platelet, lymphocyte count, total protein, albumin, prealbumin, total cholesterol, apolipoprotein E (ApoE), free fatty acid (FFA) and IL-6 were significantly different between cachexic and non-cachexic patients (all *p* < 0.05). In addition, these characteristics were different in different cancer types. When compared to male non-cachexic patients, male cachexic patients showed a significant increase in plasma levels of IL-6 and platelet, later TNM stage, with marked decrease in their plasma total protein, albumin, prealbumin, ApoE as well as their lymphocyte counts and hemoglobin levels (all *p* < 0.05). In comparison with female non-cachexic patients, female cachexic patients’ IL-6 levels and FFA were significantly elevated with noticeable decrease in their BMI, total cholesterol, ApoE and prealbumin, as well as later TNM stage (all *p* < 0.05). Correlation analysis revealed that IL-6 levels in female cachexic patients had a significant positive correlation with FFA expression, but this correlation not reflected in male patients.

**Conclusion:**

This study demonstrates the different metabolic characteristics of male and female cancer cachexia patients. Future study about cancer cachexia should pay attention to different genders and cancer types.

## Introduction

Cancer cachexia occurs in various advanced stage cancers, and approximately 80% of cancer patients may develop this state. Cancer cachexia is defined as a wasting syndrome characterized by progressive skeletal muscle atrophy and fat loss in cancer patients; it is generally accompanied by systemic inflammatory response, and cannot be fully reversed by conventional nutritional support [1]. Previous studies mainly focused on the muscle atrophy of cancer cachexia, but in recent years, more studies have shown that fat loss also plays an essential role in the occurrence and development of cancer cachexia [2]. The mechanism of fat loss mainly includes increased lipolysis, white-to-brown transdifferentiation of white adipose tissue (WAT browning), and dysfunctional adipocyte synthesis, etc. [3]. Our previous research has shown that patients with cancer cachexia lost fat mass as a result of elevated lipolysis and WAT browning [4]. Previous studies on obesity and metabolic syndromes pointed out the different characteristics of lipid metabolism in opposite genders [5, 6]. However, whether lipid metabolism differ between different sexes in cancer cachexic is unclear.

Inflammatory response is another important feature of cancer cachexia. Although previous studies showed that plasma interleukin-6 (IL-6), tumor necrosis factor-α (TNF-α) and other inflammatory factors were significantly elevated in cancer cachexia patients, there were controversies among studies [7, 8]. In particular, TNF-α expression levels in cancer cachexia patients remained a highly controversial topic. Additionally, studies conducted on animals revealed that IL-6 expressions were different in cancer cachexic mice of different genders and varied cancer types [9–11]. A deep research of IL-6 expression in opposite genders of cancer cachexic patients is needed.

In this study, we first compared the different clinical characteristics in cancer cachexia patients and non-cancer cachexia patients. Subsequently, we the compared different clinical characteristics in patients of opposite genders and different cancer types. Lastly, we analyzed the correlation between lipid metabolism parameters and IL-6 in cancer cachexia patients of different genders.

## Methods

### Patients and sample collection

Patients diagnosed at General Surgery Department of Zhongshan Hospital, Fudan University from January 2018 to June 2019 were included. The inclusion criteria were as follows: (1) Patients were pathologically diagnosed as gastric cancer or colorectal cancer; (2) Patients received radical operation; (3) Patients with complete clinical data. Exclusion criteria includes: (1) Patients received preoperative radiotherapy, chemotherapy or any other pharmacological treatments; (2) Patients accompanied by severe cardiovascular and cerebrovascular diseases, hepatic or renal dysfunction, hyperthyroidism, diabetes or HIV/AIDS; (3) Patients without complete clinical data. 5 ml of venous blood was collected from each patient preoperatively, and was subjected to centrifuge; the plasma obtained was then stored for follow-up tests.

### Patient groups

According to the international consensus of cancer cachexia dated in 2011 [1], this study divided the 311 patients into non-cachexia and cachexia groups respectively based on weight loss and Body Mass Index (BMI). The non-cachexia group is characterized by no weight loss in the past 6 months, or weight loss <5%, or BMI < 20 with weight loss of <2%; the cachexia group is characterized by weight loss in the past 6 months, with weight loss >5% or BMI < 20 accompanied by weight loss of >2%.

### Data collection

From each patient’s medical record, height, weight, gender and age were recorded. From their preoperative blood examination results, values of plasma hemoglobin, white blood cell count, platelet, lymphocyte count, total protein, albumin, prealbumin, total cholesterol, triglycerides, apolipoprotein E (ApoE), apolipoprotein B (ApoB), apolipoprotein A (ApoA) and free fatty acid (FFA) were obtained. BMI = Weight (kg) / height^2^ (m^2^). We also recorded the TNM stage of each patient according to UICC/AJCC staging system.

### Plasma measurement of inflammatory factors

The plasma concentrations of IL-6 and TNF-α were determined using a commercialized enzyme-linked immunosorbent assay (ELISA) kit (Thermo Fisher) according to the manufacturer’s protocols. Analytical sensitivity of IL-6 and TNF-α kit is 0.92 and 2.3 pg/ml, with interassay CV for 5.2 and 7.4% as well as intraassay CV for 3.4 and 6%, respectively.

### Statistical analysis

All statistical analyses were performed using GraphPad Prism 5.0, continuous data were expressed as mean ± standard error of the mean (^−^x ± sem), within the context of an independent sample t-test. Categorical data were expressed in frequencies and the χ^2^ test was adopted. The correlations of serum FFA with IL-6 in different genders of cancer cachexia patients were analyzed using Spearman rank correlation tests. Statistical significance was defined as *p* < 0.05.

## Results

### Comparison between cachexia and non-cachexia cancer patients

Among 311 patients, there were 74 cancer cachexia patients (50 males and 24 females) and 237 non-cachexia patients (150 males and 87 females). Without considering gender, in general, plasma concentrations of IL-6, FFA and platelet of patients in the cancer cachexia group were significantly higher than in the non-cachexia group. In contrast, BMI, plasma concentrations of hemoglobin, lymphocyte count, total protein, albumin, prealbumin, total cholesterol and ApoE of patients in the cancer cachexia group were significantly lower than in non-cachexia group. Furthermore, later TNM stage was more common in cancer cachexia patients (Table 1).

**Table 1:** Comparison of clinical variables between non-cachexia and cachexia patients

	Non-cachexia (*n* = 237)	Cachexia (*n* = 74)	*t/x*^*2*^	*p*
Ages (years)	62.34 ± 0.73	64.14 ± 1.19	1.226	0.221
Female no. (%)	87 (36.7%)	24 (32.4%)	0.449	0.503
BMI (kg/m^2^)	22.91 ± 0.19	22.08 ± 0.38	2.036	0.043
HB (g/L)	119.5 ± 1.68	111.8 ± 3.02	2.227	0.027
WBC (10^9/L)	5.72 ± 0.11	6.07 ± 0.25	1.422	0.156
PLT (10^9/L)	237.9 ± 5.15	262.9 ± 11.75	2.211	0.028
LY (10^9/L)	1.64 ± 0.04	1.40 ± 0.05	3.168	0.002
TP (g/L)	65.36 ± 0.41	63.64 ± 0.78	2.016	0.045
ALB (g/L)	40.88 ± 0.32	39.15 ± 0.56	2.675	0.008
PA (g/L)	0.214 ± 0.004	0.189 ± 0.006	3.525	<0.001
TC (mmol/L)	4.19 ± 0.07	3.91 ± 0.09	2.149	0.033
TG (mmol/L)	1.32 ± 0.04	1.17 ± 0.06	1.902	0.058
ApoA (g/l)	176.8 ± 12.62	220.1 ± 23.61	1.677	0.095
ApoB (g/l)	0.873 ± 0.017	0.852 ± 0.029	0.604	0.547
ApoE (g/l)	41.55 ± 0.82	36.78 ± 1.36	2.919	0.004
FFA (pg/ml)	0.42 ± 0.02	0.48 ± 0.03	2.022	0.044
IL-6 (pg/ml)	4.88 ± 0.28	8.16 ± 0.98	4.429	<0.001
TNF-α (pg/ml)	7.45 ± 0.23	8.09 ± 0.37	1.398	0.164
(I + II) TNM stage no. (%)	125 (52.7%)	24 (32.4%)	9.321	0.002

*BMI* Body mass index, *HB* Hemoglobin, *WBC* White blood cell count, *PLT* Platelet, *LY* Lymphocyte count, *TP* Total protein, *ALB* Albumin, *PA* Prealbumin, *TC* Total cholesterol, *TG* Tri-glyceride, *ApoA* Apolipoprotein A, *ApoB* Apolipoprotein B, *ApoE* Apolipoprotein E, *FFA* Free fatty acid

### Comparison in different genders between cachexia and non-cachexia cancer patients

When male cancer cachexia and male non-cachexia patients were compared, IL-6 and platelet were significantly elevated in the cachexia group, whereas total protein, albumin, prealbumin, ApoE, lymphocyte counts and hemoglobin were significantly reduced in the cachexia group. Similarly, when comparisons were made between female cancer cachexia and female non-cachexia patients, IL-6 and FFA were remarkably higher in the cachexia group, while BMI, total cholesterol, ApoE and prealbumin were apparently lower in the cachexia group. In addition, later TNM stage in cachexic patients was observed in both genders (Table 2). Interestingly, IL-6 levels were significantly elevated in both genders, but FFA was only elevated in female cancer cachexia patients.

**Table 2:** Comparison in different genders between cachexia and non-cachexia cancer patients

	male			female		
	Non-cachexia (*n* = 150)	Cachexia (*n* = 50)	*p*	Non-cachexia (*n* = 87)	Cachexia (*n* = 24)	*p*
Ages (years)	62.97 ± 0.85	65.32 ± 1.24	0.155	61.24 ± 1.33	61.67 ± 2.59	0.883
BMI (kg/m^2^)	22.82 ± 0.24	22.26 ± 0.47	0.266	23.11 ± 0.33	21.65 ± 0.67	0.047
HB (g/L)	125.9 ± 1.95	115.6 ± 3.92	0.012	108.3 ± 2.68	103.8 ± 3.99	0.416
WBC (10^9/L)	5.83 ± 0.15	6.13 ± 0.31	0.335	5.51 ± 0.18	5.92 ± 0.40	0.314
PLT (10^9/L)	232.2 ± 5.86	259.1 ± 14.07	0.039	241.9 ± 9.46	271.1 ± 21.73	0.174
LY (10^9/L)	1.61 ± 0.04	1.41 ± 0.08	0.023	1.66 ± 0.06	1.55 ± 0.09	0.376
TP (g/L)	65.18 ± 0.43	63.15 ± 0.88	0.026	66.16 ± 0.77	65.52 ± 1.72	0.715
ALB (g/L)	40.93 ± 0.36	39.3 ± 0.66	0.027	40.78 ± 0.60	38.81 ± 1.08	0.128
PA (g/L)	0.219 ± 0.005	0.186 ± 0.008	<0.001	0.199 ± 0.006	0.173 ± 0.011	0.039
TC (mmol/L)	3.97 ± 0.07	3.84 ± 0.12	0.379	4.53 ± 0.11	4.05 ± 0.17	0.043
TG (mmol/L)	1.28 ± 0.05	1.18 ± 0.06	0.235	1.35 ± 0.08	1.24 ± 0.14	0.501
ApoA (g/l)	155.2 ± 13.81	189.5 ± 23.05	0.206	187.3 ± 19.86	281.6 ± 60.36	0.057
ApoB (g/l)	0.84 ± 0.02	0.84 ± 0.04	0.905	0.94 ± 0.03	0.87 ± 0.05	0.312
ApoE (g/l)	39.37 ± 0.98	34.64 ± 1.49	0.013	46.85 ± 1.65	39.16 ± 2.28	0.028
FFA (pg/ml)	0.39 ± 0.02	0.43 ± 0.03	0.332	0.43 ± 0.03	0.56 ± 0.06	0.020
IL-6 (pg/ml)	5.23 ± 0.37	6.95 ± 0.84	0.037	4.28 ± 0.40	7.55 ± 1.25	0.001
TNF-α (pg/ml)	7.547 ± 0.283	7.654 ± 0.424	0.849	9.290 ± 0.875	8.09 ± 0.3719	0.179
(I + II) TNM stage no. (%)	76 (50.7%)	16 (32.0%)	0.022	49 (56.3%)	8 (33.3%)	0.046

*BMI* Body mass index, *HB* Hemoglobin, *WBC* White blood cell count, *PLT* Platelet, *LY* Lymphocyte count, *TP* Total protein, *ALB* Albumin, *PA* Prealbumin, *TC* Total cholesterol, *TG* Tri-glyceride, *ApoA* Apolipoprotein A, *ApoB* Apolipoprotein B, *ApoE* Apolipoprotein E, *FFA* Free fatty acid

### Comparison in different cancer types between cachexia and non-cachexia cancer patients

One hundred seventy-one gastric cancer patients and 140 colorectal cancer patients were involved in this study. Upon further dividing the groups via cancer types (gastric cancer and colorectal cancer), we found that IL-6 in gastric cancer cachexia patients was elevated significantly, while their BMI, hemoglobin, lymphocyte count, total protein, albumin, prealbumin, total cholesterol, ApoB and ApoE declined remarkably. In colorectal cancer patients, white blood cell count, platelet, ApoA, FFA, IL-6 and TNF-α increase significantly, while albumin, prealbumin and ApoE decreased significantly. Interestingly, TNM stages in cachexic patients was later than non-cachexic patients in gastric cancer but not in colorectal cancer (Table 3). The above findings indicated that there were metabolic differences between gastric cancer patients and colorectal cancer patients.

**Table 3:** Comparison in different cancer types between cachexia and non-cachexia cancer patients

	Gastric cancer			Colorectal cancer		
	Non-cachexia (*n* = 126)	Cachexia (*n* = 45)	*p*	Non-cachexia (*n* = 111)	Cachexia (*n* = 29)	*p*
Ages (years)	63.27 ± 0.92	64.34 ± 1.33	0.352	61.84 ± 1.63	62.21 ± 2.09	0.423
BMI (kg/m^2^)	23.23 ± 0.27	21.76 ± 0.47	0.008	22.71 ± 0.31	22.74 ± 0.70	0.965
HB (g/L)	121.2 ± 2.27	111.6 ± 4.36	0.038	119.2 ± 2.64	111.4 ± 4.38	0.147
WBC (10^9/L)	5.62 ± 0.15	5.51 ± 0.33	0.711	5.93 ± 0.17	7.04 ± 0.34	0.002
PLT (10^9/L)	238.2 ± 7.39	247.3 ± 15.07	0.561	241.2 ± 7.83	281.3 ± 18.35	0.023
LY (10^9/L)	1.66 ± 0.05	1.41 ± 0.09	0.012	1.58 ± 0.06	1.55 ± 0.08	0.793
TP (g/L)	65.31 ± 0.59	61.81 ± 1.03	0.004	65.67 ± 0.52	66.96 ± 1.15	0.252
ALB (g/L)	40.53 ± 0.44	38.41 ± 0.72	0.019	41.23 ± 0.46	39.10 ± 0.78	0.017
PA (g/L)	0.219 ± 0.005	0.198 ± 0.008	0.032	0.208 ± 0.006	0.178 ± 0.009	0.008
TC (mmol/L)	4.18 ± 0.09	3.74 ± 0.14	0.012	4.177 ± 0.10	4.273 ± 0.14	0.621
TG (mmol/L)	1.35 ± 0.06	1.19 ± 0.11	0.19	1.23 ± 0.06	1.32 ± 0.09	0.404
ApoA (g/l)	190.5 ± 18.68	188.2 ± 27.12	0.948	157.1 ± 16.62	277.2 ± 42.97	0.002
ApoB (g/l)	0.88 ± 0.02	0.77 ± 0.04	0.019	0.87 ± 0.03	0.97 ± 0.04	0.090
ApoE (g/l)	40.12 ± 0.99	35.74 ± 1.80	0.032	43.26 ± 1.49	37.32 ± 1.88	0.037
FFA (pg/ml)	0.41 ± 0.02	0.45 ± 0.04	0.254	0.425 ± 0.024	0.53 ± 0.05	0.041
IL-6 (pg/ml)	4.60 ± 0.37	6.82 ± 1.02	0.014	5.04 ± 0.42	7.38 ± 0.95	0.013
TNF-α (pg/ml)	7.94 ± 0.42	8.05 ± 0.63	0.894	7.32 ± 0.382	9.01 ± 0.553	0.037
(I + II) TNM stage no. (%)	70 (55.6%)	17 (35.6%)	0.023	51 (46.9%)	14 (41.4%)	0.660

*BMI* Body mass index, *HB* Hemoglobin, *WBC* White blood cell count, *PLT* Platelet, *LY* Lymphocyte count, *TP* Total protein, *ALB* Albumin, *PA* Prealbumin, *TC* Total cholesterol, *TG* Tri-glyceride, *ApoA* Apolipoprotein A, *ApoB* Apolipoprotein B, *ApoE* Apolipoprotein E, *FFA* Free fatty acid

### Comparison in cancer cachexia between male and female patients

Direct comparisons were made between male and female cancer cachexia patients, which revealed significant differences in FFA and ApoE in opposite genders (Table 4). No apparent difference was detected in other characteristics.

**Table 4:** Comparison in cancer cachexia between male and female patients

	Cachexia		
	Male (*n* = 50)	Female (*n* = 24)	*p*
Ages (years)	65.32 ± 1.24	62.83 ± 2.42	0.313
BMI (kg/m^2^)	22.26 ± 0.47	21.65 ± 0.67	0.461
HB (g/L)	115.6 ± 3.92	103.8 ± 3.99	0.067
WBC (10^9/L)	6.13 ± 0.31	5.92 ± 0.40	0.697
PLT (10^9/L)	259.1 ± 14.07	271.1 ± 21.73	0.637
LY (10^9/L)	1.40 ± 0.08	1.55 ± 0.09	0.295
TP (g/L)	63.15 ± 0.88	65.52 ± 1.72	0.179
ALB (g/L)	39.31 ± 0.66	38.81 ± 1.08	0.686
PA (g/L)	0.186 ± 0.008	0.178 ± 0.011	0.557
TC (mmol/L)	3.84 ± 0.12	4.13 ± 0.18	0.191
TG (mmol/L)	1.216 ± 0.07	1.241 ± 0.14	0.858
ApoA (g/l)	202.1 ± 25.81	246.2 ± 51.42	0.395
ApoB (g/l)	0.84 ± 0.04	0.87 ± 0.05	0.648
ApoE (g/l)	34.64 ± 1.49	41.38 ± 2.57	0.019
FFA (pg/ml)	0.43 ± 0.03	0.56 ± 0.06	0.029
IL-6 (pg/ml)	7.47 ± 1.05	7.55 ± 1.25	0.961
TNF-α (pg/ml)	7.65 ± 0.42	8.74 ± 0.71	0.171
(I + II) TNM stage no. (%)	16 (32.0%)	8 (33.3%)	0.909

*BMI* Body mass index, *HB* Hemoglobin, *WBC* White blood cell count, *PLT* Platelet, *LY* Lymphocyte count, *TP* Total protein, *ALB* Albumin, *PA* Prealbumin, *TC* Total cholesterol, *TG* Tri-glyceride, *ApoA* Apolipoprotein A, *ApoB* Apolipoprotein B, *ApoE* Apolipoprotein E, *FFA* Free fatty acid

### Correlation analyses between plasma IL-6 and lipid metabolism parameters in cachexia patients of different genders

We focused on analyzing the correlation of IL-6 and lipid metabolism parameters, and discovered a positive correlation between plasma IL-6 and FFA in female cancer cachexia patients, but no significant correlation were observed in male patients (Table 5).

**Table 5:** Correlation analyses between plasma IL-6 and lipid metabolism parameters in cachexia patients of different genders

	Male		Female	
	r	*p*	r	*P*
TC	−0.19	0.247	0.02	0.933
TG	−0.19	0.248	−0.09	0.698
ApoA	0.08	0.632	0.06	0.805
ApoB	−0.10	0.543	0.35	0.157
ApoE	−0.23	0.168	−0.23	0.357
FFA	−0.19	0.24	0.47	0.037

*TC* Total cholesterol, *TG* Tri-glyceride, *ApoA* Apolipoprotein A, *ApoB* Apolipoprotein B, *ApoE* Apolipoprotein E, *FFA* Free fatty acid

## Discussion

Cancer cachexia generally accompanied by systemic inflammatory response and fat loss. Previous studies mainly divided cachexia patients by degree of weight loss, but seldom consider the differences between genders. This study, through analyzing 311 gastric cancer and colorectal cancer patients, found that the inflammatory factor, IL-6, was significantly elevated in male and female patients, but TNF-α, another common inflammatory factor concerning cachexia, had no apparent change in both male and female patients, indicating that serum IL-6 might play a more important role during the progress of cachexia. In addition, FFA was significantly elevated in female cancer cachexia patients only, which strongly suggests that male and female cancer cachexia patients have metabolic differences.

In this study, we first compared the differences in clinical data between cancer cachexia and non-cachexia patients, regardless of gender; albumin, prealbumin, lymphocyte count and other conventional nutritional markers decreased in cachexia patients. Incidentally, we found that blood platelet levels in cancer cachexia patients were significantly elevated, which might be associated with the “first responder” properties of platelet during chronic inflammation, cancer progression and metastasis [12]. However, the detailed mechanism remains to be determined. Upon further investigating according to genders, we found distinct differences of many markers in male and female cachexia patients compared with non-cachexia patients. These findings suggested that different genders should be separately analyzed in future investigation regarding the cancer cachexia.

Previous studies mainly emphasized on muscle atrophy and its mechanism during cancer cachexia. Our previous study also revealed that inflammatory factors promoted muscle loss in cancer cachexia patients [13]. However, recent studies have revealed that fat loss might play a crucial role in the progress of cancer cachexia [7]. In this study, we incidentally found that plasma concentration of ApoE significantly decreased in cancer cachexia patients, which was the first report as far as we known. ApoE a component of chylomicrons (CM), very low-density lipoproteins (VLDL), as well as the remnants and β-VLDL. The concentration of ApoE showed positive correlation with plasma triglyceride level [14]. ApoE genotype has the most profound genetic risk on late onset Alzheimer’s disease and also affects processes in normal brains [15, 16]. In Alzheimer’s disease, ApoE interactions with molecules important for lipid efflux and lipid endocytosis underlie effects of ApoE genotype on neuroinflammation and lipoprotein composition [15]. The role of ApoE in cancer cells is unclear and limited studies have addressed it. ApoE has been found to be related to lung cancer and considered to be a useful marker for assessing NSCLC patients with lymph node metastasis [17]. ApoE was also recognized as new pancreatic cancer biomarker but had no association with risk of prostate cancer [18, 19]. The role of ApoE in cancer cells is unclear except that ApoE promoting cancer cell proliferation and migration [20, 21]. In our study, plasma concentration of ApoE was downregulated in cancer cachexia patients compared to non-cancer cachexia patients regardless of genders and cancer types, indicating its important role during the progress of cancer cachexia. However, further study is needed to investigate the role of ApoE in cancer cachexia.

FFA is the primary fuel for heart and skeletal muscles and are precursors of hormones and non-hormonal signalling molecules. FFA plays important physiological roles in skeletal muscle, heart, liver and pancreas. However, chronically elevated plasma FFA appear to have pathophysiological consequences [22]. Elevated FFA was linked with the onset of insulin resistance and associated with type-2 diabetes, by inhibiting the tyrosine phosphorylation of IRS-1 (insulin receptor substrate-1) and reducing IRS-1-ssociated PI3K (phosphatidyl-inositol 3-kinase) activity which is responsible for transducing downstream insulin signals [23, 24]. In this study, we screened out the influences of diabetes, hyperthyroidism and other related diseases to investigate the association of FFA and cancer cachexia induced lipolysis. When we studied cancer cachexia patients according to gender, we discovered that FFA increased significantly in female patients only, while male patients demonstrated an elevating trend. Studies have reported greater serum FFA concentrations in estrogen-deficient women compared with postmenopausal women receiving estrogen treatment, suggesting that estrogen treatment has beneficial effects in postmenopausal women by preventing the accelerated delivery of FFA into the circulation [25]. Estrogen may attenuate lipolysis occurring in adipose tissue through the up-regulation of α2-adrenergic receptors, resulting in decreased mobilization of FFA from adipose tissue [26]. Estrogen also regulated the FFA release by regulating the hormone sensitive lipase (HSL) and lipoprotein lipase (LPL) [27]. In our study, 5 out of 24 female (20.8%) cachexic patients and 15 out of 87 female (17.2%) non-cachexic patients were less than 50 years old, indicating the estrogen reduction might have a similar effect on the FFA in both groups. Therefore, the elevated concentration of FFA in female cachexic patients might be attribute to the abnormal metabolism of fat tissues and further study should be conducted to analyze the specific mechanisms.

Studies have shown that inflammatory factors (especially IL-6 and TNF-α) play an important role in the occurrence and development of cancer cachexia [8]. In animal cachexia models, both plasma IL-6 and TNF-α were significantly increased [28]. However, the serum results of cancer cachexia patients were controversial [29], and that inhibiting TNF-α in cancer cachexia patients with weight loss did not halt the process of muscle loss in patients [30]. TNF-α is one inflammatory mediator that has been implicated in carcinogenesis, due to its participation in chronic inflammatory diseases [31]. There is controversy; however, regarding the role of TNF-α in cancer, the pro- or antitumoral TNF-α response with in the tumor microenvironment depends not only on local concentration but also on its expression site in the tumor [32]. Studies have reported the involvement of TNF-α in lipolysis by decreasing LPL activities [33]. TNF-α was also a potent inhibitor of lipogenesis and suppressed the expression of gene responsible for various nuclear transcription factors that were required for differentiation and function of adipocyte [33]. These findings indicate the role of TNF-α in adipose tissue loss and in the development of cancer cachexia [34]. Our study showed that no apparent changes of plasma TNF-α in cancer cachexia patients were observed in both genders. However, TNF-α was increased in colorectal cancer cachexia patient, indicating that TNF-α might play a role in the development of cancer cachexia in colorectal cancer cachexia but not in gastric cancer cachexia.

Conversely, serum IL-6 level was significantly elevated in both male and female cancer cachexia patients, which strongly implied that IL-6 plays an important role in the progress of cancer cachexia. Previous studies implicated IL-6-STAT3 in fat wasting and the acute phase response in cancer cachexia [35]. We found a significant positive correlation between IL-6 and FFA levels in female cancer cachexia patients but no correlation was observed in male cancer cachexia patients. This finding suggests that IL-6 may be a primary factor in promoting lipolysis in female patients with cancer cachexia, resulting in elevated FFA.

### Limitations

The main limitation of this study was a relatively limited sample size. Only 24 females cancer cachexia patients were included and when we conducted subgroup analysis might cause the potential bias. We will include more patients to validate the results in further studies. Furthermore, only two cancer types (gastric cancer and colorectal cancer) are investigated in this study and whether other cancer types such as pancreatic cancer showed the similar characteristics is not convinced. We will include more patients with different cancer types in further studies.

## Conclusion

In conclusion, by analyzing the differences in male and female cancer cachexia patients, we discovered different metabolic characteristics between each gender. In addition, patients with different cancer types also showed different metabolic characteristics. Plasma IL-6 was significantly elevated in both genders, but FFA was only elevated in females and positively associated with IL-6. Our findings from this research serve as a reminder in the future to study and treat cancer cachexia patients with respect to their gender and cancer types.

## Data Availability

The datasets during and/or analyzed during the current study available from the corresponding author on reasonable request.

## Abbreviations

ALB: Albumin
ApoA: Apolipoprotein A
ApoB: Apolipoprotein B
ApoE: Apolipoprotein E
BMI: Body mass index
FFA: Free fatty acid
HB: Hemoglobin
LY: Lymphocyte count
PA: Prealbumin
PLT: Platelet
TC: Total cholesterol
TG: Tri-glyceride
TP: Total protein
WBC: White blood cell count
